# Labelling fish diets with ^15^ *N* ‐Leucine for monitoring feed consumption and bio‐distribution in Atlantic salmon

**DOI:** 10.1002/vms3.730

**Published:** 2022-03-29

**Authors:** Mirta Cortés‐Quezada, Ana María Parada, Ximena Videla, Juan Antonio Valdés, Sebastián Gonzalez‐Catrilelbún, Alexis Aspée, Adriana Nario, Andrea Rivas‐Aravena

**Affiliations:** ^1^ Facultad de Medicina y Ciencia Universidad San Sebastián Providencia Santiago Chile; ^2^ Departamento de Tecnologías Nucleares Comisión Chilena de Energía Nuclear Las Condes Chile; ^3^ Facultad de Ciencias de la Vida Laboratorio de Biotecnología Molecular Universidad Andrés Bello Santiago Chile; ^4^ Facultad de Química y Biología Departamento de Ciencias del Ambiente Universidad de Santiago de Chile Santiago Chile

**Keywords:** feeding in aquaculture, fish feeding, food marker, nitrogen biodistribution, oral intake

## Abstract

Feeding represents 50–70% of the cost of production in salmon farming, higher than any other animal farm. The improvement of this percentage is challenging as the food is thrown into the fish tank, there is no quantification of the amount of food that is consumed by the fish. In consequence, it is difficult to adjust the food composition making it more nutritive or promoting food consumption by fish. In this study, to investigate food consumption, bio‐distribution and food residues, leucine containing ^15^N (a stable isotope of nitrogen) was used to label the fish food. Atlantic salmon (*Salmo salar*) weighing 100–120 g were maintained in 30 L tanks at a density of 14 kg/m^3^. Fishes were fed daily at 1% of the fish weight with pellet labelled with ^15^ *N*‐leucine. The ^15^ *N* incorporation was determined 14 hours after the feeding in all the fish organs. Results showed that 14 hours after the administration of a single dose of labelled food to Atlantic salmon enables the detection of the tracer in the whole organism allowing determining the food consumption. Through the analysis of nitrogen use efficiency (NUE), we showed that the trunk, pyloric caeca and head incorporate the highest level of the marker (72.7, 8.7 and 5.7%, respectively). This methodology would permit monitoring feeding to minimize food loss, improve administration methodologies or select the preferred foods for the fish, among others to reduce production costs.

## Graphical abstract

Administration of the labelled food to fish allows knowing the real consumption of food by fish. It is possible to determine the distribution and metabolism of nitrogen coming from the labeled amino acids. The marker added to the food can be detected since hours post feed, until its complete elimination form fish, with high sensitivity.

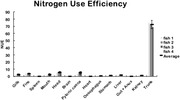

## 1 INTRODUCTION

The human population is overgrowing and is predicted that in 2060, it will exceed nine billion inhabitants. In this scenario, aquaculture development in coherence with an environmentally sustainable, socially fair manner to provide healthy and safe products, is visualized as an ideal solution. Despite that, there are still severe limitations on fish production to make it accurate. Although the aquaculture has been developed faster than any other sector of the food industry of animal origin, one of the weaknesses is that 50–70% of the cost of fish production is feeding, beyond any other animal products. Additionally, it is difficult to determine the amount of food administered that is consumed by fish, avoiding diminishing this cost of production (Asche & Oglend, 2016).

Currently, the feed efficiency is determined by calculating a feed conversion ratio (FCR) that establishes the relation between the quantity of food administered and the fattening of the fish. Nevertheless, the feeding of fish is variable depending on the development and sanitary status of fish, temperature, population density, oxygenation, gastrointestinal microbiota and salinity of the medium (Bett et al., 2016; Bou et al., 2017; Furey et al., 2016) causing that FCR is different for each fish farming centre.

To improve the FCR of the salmon, it is required to adjust (i) the nutrients composition of the food, optimizing the feeding efficiency, and/or (ii) the food consumption, related to the feeding method, and characteristics of the food (palatability, time of flotation, among others) (Hasan & Soto, 2017; Kamunde et al., 2019; Rosenlund et al., 2016; Weihe et al., 2018l). It is required to determine the food intake for optimizing both strategies.

Actually, some methodologies have been used to trace food and to study intestinal transit utilizing inert, non‐metabolizable and non‐absorbable markers not interfering with absorption, digestion or intestinal transit, such as chromium oxide, ^141^Cerium, crystal beads, titanium oxide, iron particles, radio‐opaque glass beads or radioactive elements like^131^Iodine. The digestibility of the food is determined by quantifying the marker in the food and in faeces (Abowei & Ekubo, 2011; Degani et al., 1997; Kause et al., 2006). These methodologies do not track consumed food, limiting them to the determination of apparent consumption of food.

In particular, the measure of protein digestibility is frequently correlated with the nitrogen (N) in the food and the N excreted for the fish. However, the N excreted also comes from the metabolism of the fish, and for this quantification, a protein‐free diet as a control is required; however, this diet alters the nutrition of the fish and the results (Carter et al., 1998; Carter & Hauler, 2000; Kaushik & De Oliva Teles, 1985; Kaushik et al., 1983).

On the other hand, the distribution of proteins in living organisms has been estimated by monitoring ^15^N as a common tracer element based on that ^15^N represents around 0.366% of the total element abundance (Faust & Sebastianelli, 1987). In fact, taking advantage of that, ^15^N has been externally added to nutrients, fertilizers or growth media in plants (Nario et al., 2003), bacteria (reviewed in (Gtari et al., 2012) and mammals (discussed in [Duggleby & Waterlow, 2005]) to determine bio‐distribution and clearance of nutrients and metabolites in the organism labelled with ^15^N (Bos et al., 1999, De Preter et al., 2004; Moughan et al., 1998; Nario et al., 2003; Weijs et al., 1996). In particular, in aquaculture, proteins labelled with ^15^N protein have been administered to gilthead seabream (*Sparus aurata*) (Felip et al., 2012), Atlantic halibut (*Hippoglossus hippoglossus*) (Fraser et al., 1998) and rainbow trout (*Oncorhynchus mykiss*) (Beltrán et al., 2009) to evaluate protein bio‐distribution. Besides, diets enriched with ^15^N have been used to study protein synthesis in fish (Carter et al., 1994).

In this work, we propose to use the essential amino acid leucine enriched with^15^N as a marker in the diet to investigate the intake, absorption, bio‐distribution and evacuation of administered food (Bogatyreva et al., 2006). ^15^N‐leucine will be digested and absorbed by the fish, allowing them to trace it and quantify its metabolism in tissues and organs in time.

## 2 MATERIALS AND METHODS

### 2.1 Fish maintenance

The experiments were performed in Atlantic salmon (*Salmo salar*) weighing 100–120 g (20–23 cm). Fishes were kept in freshwater tanks of 30 L at a density of 14 kg/m^3^, a temperature of 15°C, and an oxygen rate of 8–9.5 mg/L (Rivas‐Aravena et al., 2015). Fishes were fed manually at 13:00 hours. During the acclimatization period, it will reach a feeding regime of 1% of the fish weight.

### 2.2 Labelling food

The food was mixed with 0.5 mL of a solution of 0.18 M ^15^N‐leucine (Sigma‐Aldrich) and 0.3 mL of vegetable oil per gram of a commercial pellet (size 50, Ewos, for composition, see Table 1). The ^15^N‐leucine food was dried for 24 hours at 30°C.

**TABLE 1:** Diet composition

Ingredients	Content(%)
Crude protein	48
Lipids	20.5
Humidity	11
Cenizas	11
Crude Fibre	2.5

Ingredients used in the food formula: Fish meal, fish oil, vegetable oil, soy lecithin, corn protein concentrate, soy protein concentrate, soy and/or derivatives, blood meal/hemoglobin, wheat gluten, sunflower meal, poultry by‐products, wheat and/or derivatives, binders, betaine, inorganic phosphate, corn derivative, full vitamin premix, mineral premix, methionine, lysine, antioxidants, astaxanthin, vitamin C monophosphate and pea or pea derivative

### 2.3 Feeding

Previous studies of the food digestion in salmonids and other teleost indicate that the stomach is filled about 5 hours after feeding and remained empty about 14 hours after feeding. The intestine content peaked between 12 and 14 hours after feeding. These parameters depend on the amount and composition of the diet, the absorption of nutrients, and environmental conditions such as temperature (Aas et al., 2017; Bravo et al., 2018; Magnuson, 1969; Sveier et al., 1999). We choose to perform the analysis at 14 hours after feeding to ensure that the food had left the stomach and the nutrients have been absorbed in the intestine.

To set the time of food digestion, fishes were fed for 1 or 5 days with ^15^N‐leucine‐labeled food in a proportion of 1% of the fish weight (two fishes per group). No consumed food was collected 30 min after feeding and faeces were collected daily using a fish net. Fourteen hours after the last feeding, fishes were sacrificed by immersing them in an excess of Benzocaine.

Once it was known, the proper time for marker detection on fish tissues, fishes (n = 4) were fed once, and 14 hours later they were sacrificed for the analysis.

### 2.4 Sampling

The fish was dissected in gills, fins (including dorsal, caudal, pectoral, anal and ventral fins), mouth, head, brain, spleen, heart, liver, kidney, oesophagus, pyloric caeca, stomach, gut + anus and trunk. The trunk corresponds to the muscular tissue from the operculum until the caudal fin including the skin, vertebral column, vertebral arches and ribs.

### 2.5 Total nitrogen and ^15^N determination

Total nitrogen (Nt = ^14^N + ^15^N) was quantified in the samples by the Kjeldahl method (Kjeldahl, 1883) and ^15^N was quantified by optical spectroscopy emission (Faust & Sebastianelli, 1987), with an emission spectrometer ^15^N analyser NOI‐6 PC as has been described (Nario et al., 2003). The content of ^15^N was reported in units of ^15^N atom percent excess (atom % excess ^15^N). In this methodology, all ^15^N enriched is quantified over the background, not requiring a negative control.

### 2.6 Calculation

Nitrogen Use Efficiency (NUE) was determined as has been described (Zapata, 1990).

$$\begin{array}{c} \mathrm{NUE}\;=\;100\;\times \;\mathrm{Nddf}\;\left(\mathrm{mg}\right)\;\text{in}\;\text{tissue}/\text{Nt}\;\text{in}\;\mathrm{administered}\;\text{food} \end{array}$$

where

$$\begin{array}{ccc} & & Nddf\;=\\ & & \;\;\;\;\left(\mathrm{atom}\;\%\;\mathrm{excess}\;{}^{15}N\;\text{in}\;\text{tissue}\right)/\left(\mathrm{atom}\;\%\;\mathrm{excess}\;{}^{15}N\;\text{in}\;\text{labelled}\;\text{food}\right). \end{array}$$

An example of the calculation of NUE is shown in the Supporting information.

### 2.7 Statistics

Statistical analysis of NUE result was performed with Kruskal–Wallis One‐way ANOVA on rank tests followed by post‐hoc Dunn's multiple comparison tests. Differences with a *p*‐value lower than 0.05 were considered significant. All the statistical analysis was conducted on Prism software.

## 3 RESULTS

### 3.1 Evaluation of ^15^N incorporation in fish tissues on time

**FIGURE 1:**
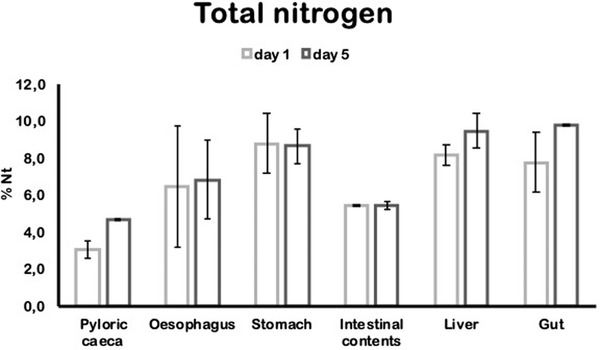
Total nitrogen content in tissues from the gastrointestinal tract and intestinal contents. Fish fed ^15^N‐leucine food for 1 or 5 days. Total nitrogen content was quantified in pyloric caeca, oesophagus, stomach, liver, gut and intestinal contents by the Kjeldahl method (n = 2)

To establish the experimental conditions to perform the analysis of food intake, a first experiment was planned for determining when the marker was incorporated and detected in the fish's gastrointestinal tract, for studying afterwards the bio‐distribution of the marker on the whole fish. For this, only two fishes, Atlantic salmon (100 g) were fed once a day for 1 or 5 days with ^15^N‐leucine‐ labelled food and the absorption of ^15^N‐leucine in the gastrointestinal tract was analyzed after 14 hours from the last feeding. This analysis was performed with two fishes per treatment for timely detection of the tracer into the organism required to evaluate its bio‐distribution in subsequent studies. Nt content was determined in the gastrointestinal tract: gut, pyloric caeca, oesophagus, stomach and liver. Additionally, Nt was quantified in intestinal contents and excreted faeces. In Figure 1, it is observed that the liver, gut and stomach contain more Nt than oesophagus and pyloric caeca, and the content of Nt in tissues from the gastrointestinal tract and the intestinal contents remain constant between the first to the fifth day.

Detection of ^15^N in the gastrointestinal tract shows that there was no difference in ^15^N incorporation in fish between days 1 and 5 (Table 2). On day 5, the content of ^15^N varied between both fishes, the higher content in fish 4 is in agreement with a higher consumption of food, in fact, the gut of fish 4 was filled with food, while fish 3′s intestine was empty. These results show that ^15^N was mainly accumulated in the pyloric caeca and stomach, followed by liver and intestine; ^15^N was detected in intestinal contents but not in excreted faeces, indicating that ^15^N was utterly absorbed by the fish (data not shown).

**TABLE 2:** ^15^N content on the gastrointestinal tract and intestinal contents of fish

	Day 1		Day 5	
Atom % excess ^15^N	Fish 1	Fish 2	Fish 3	Fish 4
Pyloric caeca	0.041	0.062	0.073	0.225
Oesophagus	0.039	0.056	0.039	0.053
Stomach	0.057	0.055	0.046	0.176
Intestinal contents	0.035	0.071	0.058	0.163
Liver	0.036	0.056	0.066	0.158
Gut	0.020	0.036	0.047	0.154

Fish fed ^15^N‐leucine food during 1 or 5 days. Fishes were dissected in pyloric caeca, oesophagus, stomach, liver and gut. Intestinal contents were also analyzed. ^15^N was determined by optical spectroscopy emission and reported as atom % excess ^15^N (n = 2).

These results indicate that it is possible to detect the tracer in the gastrointestinal tract after 14 hours of feeding with labelled food, indicating that the bio‐distribution of the tracer in the fish can be evaluated only after one feeding dose. Then, 14 hours after the fishes were fed once, the marker bio‐distribution was analyzed in all their tissues and NUE was determined.

### 3.2 Analysis of NUE in fish organs after 14 hours of feeding labelled food

Fishes were fed once with ^15^N‐leucine labelled food and dissected after 14 hours, quantifying N content in gills, fins, mouth, head, brain, spleen, heart, liver, kidney, oesophagus, pyloric caeca, stomach, gut + anus and trunk.

**FIGURE 2:**
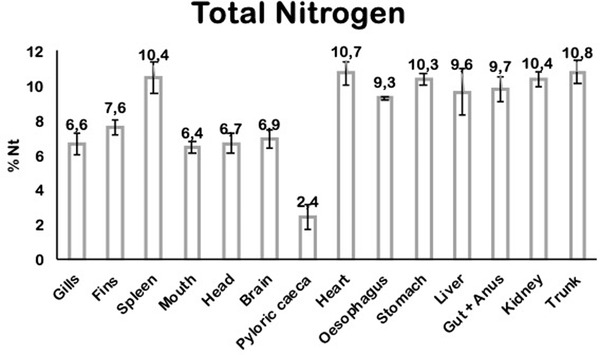
Quantification of total nitrogen in fish 14 hours after feeding ^15^N‐leucine food. Total nitrogen content was quantified by the Kjeldahl method in each tissue from fish. Standard deviation is shown (n = 4)

The Nt content in the spleen, heart, stomach, kidney, trunk, gut + anus, liver and oesophagus are the highest, while pyloric caeca had the lowest content of Nt (Figure 2).

The quantification of ^15^N atom in excess (%) in each tissue is shown in Table 3. It is noteworthy that ^15^N above natural abundance was found in all tissues of the fish after 14 hours of feeding. The percentage of the ^15^N from the total N was higher in the first portion of the intestinal tract (stomach, pyloric caeca, oesophagus) followed by the liver and kidney. ^15^N was detected in a minor proportion in gills, mouth, spleen, heart, brain, head, fins and trunk, and gut + anus shows the lowest ^15^N content.

**TABLE 3:** Atom % excess ^15^N present on every tissue from fish

Atom % excess ^15^N ± SD	
Gills	0.039 ± 0.011
Fins	0.026 ± 0.005
Spleen	0.038 ± 0.009
Mouth	0.034 ± 0.020
Head	0.027 ± 0.006
Brain	0.031 ± 0.006
Pyloric caeca	0.063 ± 0.010
Heart	0.047 ± 0.011
Oesophagus	0.048 ± 0.002
Stomach	0.071 ± 0.023
Liver	0.055 ± 0.005
Gut + Anus	0.024 ± 0.004
Kidney	0.053 ± 0.007
Trunk	0.021 ± 0.002

Quantification of ^15^N in fish 14 hours after feeding ^15^N‐leucine food, by optical spectroscopy emission. Standard deviation is shown (n = 4).

The ^15^N atom excess (%) does not allow estimating the ^15^N‐leucine‐food bio‐distribution in the fish by itself. It is required to relate the ^15^N atom excess (%) with the total N content and the tissue weight to calculate the NUE (Zapata, 1990), which is based on the mass balance principle (EU‐Nitrogen Expert Panel, EU Nitrogen Expert Panel, 2015). An example of the calculation of NUE is shown in Supporting information. The NUE values show the distribution of ^15^N acquired from the food in each tissue. The summation of the NUE of each organ permits to calculate total NUE, representing the percentage of food consumed by the fish. Total NUE was similar for each fish (fish 1: 67.1 %; fish 2: 57.3 %; fish 3: 63.5% and fish 4: 61.4 %) with an average of 62.5% of ^15^N‐leucine food consumption.

**FIGURE 3:**
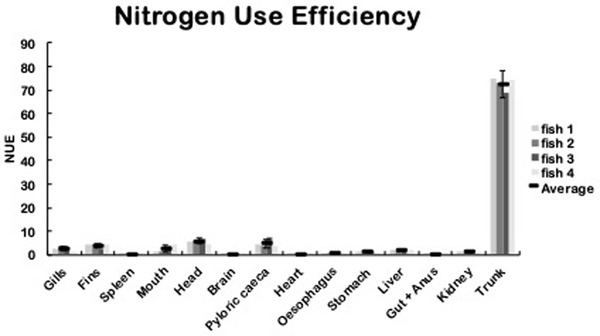
Nitrogen use efficiency on each tissue from fish. NUE (^15^N yields per ^15^N‐leucine food consumed) was determined in each tissue 14 hours after feeding ^15^N‐leucine food (columns). The average NUE and the standard deviation are shown (n = 4). Statistical analysis was performed with Kruskal–Wallis One‐way ANOVA on rank tests followed by post‐hoc Dunn's multiple comparison tests. *p < 0.01 was considered a significant change

The distribution of ^15^N was similar in tissues for every fish. From total ^15^N consumed for fish, the trunk accumulated an average of 72.7% (Figure 3). In the gastrointestinal tract, 8.7 % of ^15^N is accumulated mostly in pyloric caeca, mouth and stomach, and to a lesser extent in gut + anus. Also, the NUE in the oesophageal, stomach, and intestinal content and the faeces was 0.2, 0.9, 1 and 0%, respectively (Table 4). This result indicates that all ^15^N present in food was absorbed in the gastrointestinal tract, validating the efficiency of ^15^N‐leucine as a marker of food consumption and absorption in fish.

**TABLE 4:** Nitrogen use efficiency for every tissue from fish

Nitrogen use efficiency	
Gills	2.360 ± 0.857
Fins	3.639 ± 0.975
Spleen	0.152 ± 0.036
Mouth	2.695 ± 1.396
Head	5.671 ± 1.207
Brain	0.164 ± 0.0460
Pyloric caeca	4.663 ± 1.880
Heart	0.265 ± 0.048
Oesophagus	0.7709 ± 0.067
Stomach	1.324 ± 0.444
Liver	1.830 ± 0.152
Gut + Anus	0.180 ± 0.020
Kidney	1.436 ± 0.125
Trunk	72.707 ± 5.718

NUE was determined according to supplementary information. Standard deviation is shown (n = 4)

Apart of the intestinal tract, ^15^N mostly accumulates on the head (5.7 %), fins (3.7%), gills (2.4%), liver (1.8%) and kidney (1.4%), and in a less proportion, ^15^N is detected in heart (0.3%), brain (0.3%) and spleen (0.2%) (Figure 3, Table 4). This result shows that it is possible to determine the bio‐distribution and metabolization of ^15^N‐leucine in fish.

All these results demonstrate that ^15^N isotope can be used to quantify the intake of food in fish and the bio‐distribution of a labelled amino acid in different tissues in the fish after only one dose of feed.

## 4 DISCUSSION

In this research, ^15^N‐leucine‐labeled food was used to feed fish to quantify food consumption, and incorporation and bio‐distribution of labelled amino acid. The results showed that this methodology allows analysing the food intake after only one administration of food. Indeed, the gastrointestinal tract distribution of ^15^N is almost the same on day 1 or 5 after feeding.

Nt was quantified in each organ and tissue, responding to the protein content in the tissue and its function. For example, the pyloric caeca, brain and head showed the lower Nt content in agreement with the high proportion of fat in these tissues. Trunk comprises the higher quantity of Nt because it is constituted mainly of muscular tissue. Similarly, the high amount of Nt in the heart, spleen, kidney and stomach correlates with its high content of the muscular and connective tissue.

Elevated levels of ^15^N were present in all tissues of the fish. This detection indicates that ^15^N‐leucine was rapidly absorbed after food consumption. Indeed, it was achieved that all ^15^N‐leucine was adsorbed in the gastrointestinal tract, and not in excreted faeces.

NUE relates the ^15^N content in each tissue with its total N and weight; consequently, NUE value denotes (i) the ^15^N uptake efficiency, which is the ability of fish to take up ^15^N from food, and (ii) the use efficiency of the absorbed ^15^N, that is the efficiency of each tissue to use the absorbed ^15^N (Bos et al., 1999; Cantalapiedra‐Hijar et al., 2018; Congreves et al., 2021; De Preter et al., 2004; Moughan et al., 1998; Nario et al., 2003; Weijs et al., 1996; Zapata, 1990). Regarding the ^15^N uptake efficiency, NUE shows an average of 62.5% ^15^N incorporated in the fish, indicating that 62.5% of food was consumed.

Concerning the use efficiency of the absorbed ^15^N, organs containing a high proportion of Nt and weight will have higher NUE values compared with organs of low weight and Nt, regardless of their atom % excess ^15^N, thus, ^15^N is distributed in decreasing order on the trunk, head, pyloric caeca, fins, mouth, gills, liver, kidney, stomach, oesophagus, heart, gut + anus, brain and spleen. The detection of ^15^N on the first portion of the gastrointestinal tract (mouth, pyloric caeca and stomach) denotes a rapid absorption of ^15^N‐leucine after the consumption before the alimentary bolus arrived at the oesophagus. On the other hand, the high NUE value in the pyloric caeca could be because this organ has a broad absorption surface.

The absorbed ^15^N‐leucine gets into the bloodstream, where they can be taken up by all cells of the body. The branched‐chain Leu enters into the cell mediated by transporters (Brosnan & Brosnan, 2006). Once inside the organs and tissues, the ^15^N‐leucine can follow an anabolic pathway forming a part of new proteins or a catabolic pathway to generate energy. In ammoniotelic teleost, amino acid catabolism occurs in the liver, kidney, muscles and gills. The first product of amino acid degradation is ammonium, which is excreted through the branchial epithelium (Kaushik et al., 1983). These antecedents are congruent with that of liver, kidney, muscles, and gills show high NUE values, the trunk being the area of highest deposition of ^15^N‐leucine.

Leucine passes through the blood–brain barrier to participate in the production of brain neurotransmitters. In the cell to produce glutamate, the leucine NH_3_ group is transferred to alpha‐ketoglutarate (Erecińska & Nelson, 1990; Yudkoff, 1997). Consequently, ^15^N detection in the brain agrees with its high leucine and glutamate content. Also, ammonia can be accumulated in the brain of ammoniotelic fish, contributing to the detection of ^15^N in the fish brain (reviewed in Chew et al., 2005; Ip et al., 2004).

Since the measurement was made at early feeding times, the branchial and urinary excretion of ammonia was not achieved. However, the gills show an NUE value higher than other organs. This could be explained by the detection of the beginning of ammonia excretion following the ^15^N‐leu metabolism (reviewed in Wilkie, 2002), since some reports show that 40–60% of the nitrogen intake from food is excreted within 24 hours (Ip et al., 2004; Lim et al., 2001).

The use of ^15^N to determine the ileal endogenous amino acid digestibility has some limitations in birds and pigs. In birds, it has been shown that results of using ^15^N delivered by intravenous infusion depend on various factors such as the intake of anti‐nutritional agents, age, the diet itself, the administration way, among others (Soomro et al., 2017). In pigs, the ^15^N‐leucine infusion technique may overestimate the ileal endogenous nitrogen losses (Leterme et al., 1998). Nevertheless, the information provided for quantifying food consumption in fish will be valuable for testing food preference, developing strategies for minimizing food losses and improving administration methodology. In this way, the aquaculture companies will be able to reduce their food expenses, improving management techniques, while the manufacturing companies will be able to optimize the food, generate more palatable food for the fish or adjusting the nutrients. In particular, information on the changes in the intake of food in different conditions during production would improve the flattering in the fish production.

Moreover, the use of ^15^N is not restricted to the quantification of food but any oral treatment applied on aquaculture and for any aquatic organism that could be farmed. This versatile tool will allow improving many areas of aquaculture production, making this industry more sustainable.

## 5 CONCLUSIONS

In conclusion, our results show that to trace the food in fish, ^15^N can be used as a tool to evaluate the oral intake of food in fish, quantifying the effective ingestion and absorption by the fish. It was demonstrated that food labelled with ^15^N‐leucine is absorbed as soon as 14 hours after feeding, and that bio‐distribution shows that the tracer accumulates in the trunk, head, pyloric caeca, fins, mouth, gills, liver, kidney, stomach, oesophagus, heart, gut + anus, brain and spleen.

## ETHICAL STATEMENT

The authors confirm that the ethical policies of the journal, as noted on the journal's author guidelines page, have been adhered to and the appropriate ethical review committee approval has been received. The authors confirm that they have followed EU standards for the protection of animals used for scientific purposes.

## AUTHOR CONTRIBUTION

The authors confirm contribution to the paper as follows: study conception and design: AR‐A, AN; data collection: MC‐Q, AMP, XV; analysis and interpretation of results: MC‐Q, SG‐C, JAV, AA, AR‐A, AN; draft manuscript preparation: AR‐A, AA. All authors reviewed the results and approved the final version of the manuscript.

## FUNDING INFORMATION

This research was financed by the Proyecto semilla n° 675 from the Comisión Chilena de Energía Nuclear.

### PEER REVIEW

The peer review history for this article is available at https://publons.com/publon/10.1002/vms3.730

## Supporting information

Supporting information

## Data Availability

The datasets generated during the current study are available from the corresponding author on reasonable request.
