# Supplementary material for: Labelling fish diets with 15 N ‐Leucine for monitoring feed consumption and bio‐distribution in Atlantic salmon
Source: Vet Med Sci. 2022 Mar 29;8(3):1096–103. doi: 10.1002/vms3.730 (PMC9122439; doi:10.1002/vms3.730)
Supplement: Supplementary file 1 — Supporting information [file VMS3-8-1096-s001.pdf]

Example of calculation of Nitrogen Use Efficiency (NUE) in gills and in total fish

Data
Sample: Gills
Total mass = 599.8
Sample mass = 100.5 mg
Nt in sample = 6.6 mg
% Nt = 6.6 %
% ^{15}N excess in sample: 0.047% atom % ^{15}N
Administered labelled food-^{15}N = 1 g
% ^{15}N excess in labelled food= 1.5 atom % ^{15}N
Nt in administered labelled food = 74.66 mg

Nitrogen derived from food (Ndff) in the sample from gills

$$Nddf\ (\%) = \frac{\%\ 15\text{N excess in sample x } 100}{\%\ 15\text{N excess in labelled food}}$$

$$Nddf\ (\%) = \frac{0.047 \text{ x } 100}{1.5} = 3.2\%$$

$$Nddf\ (mg\%) = \frac{\text{Nt in sample (mg) x Ndff } (\%)}{100}$$

$$Nddf\ (mg\%) = \frac{6.6 \text{ x } 3.2}{100} = 0.21\ mg$$

NUE in gill

$$\text{Total Nitrogen in gill (mg)} = \frac{\text{Total mass gill (mg) x Total Nitrogen in Sample (mg)}}{mass\ sample\ (mg)}$$

$$\text{Total Nitrogen in gill (mg)} = \frac{599.8 \text{ x } 6.6}{100.5} = 39.4$$

$$\text{Ndff (mg)} = \frac{\text{Total Nitrogen in gill (mg) x Ndff } (\%)}{100}$$

$$\text{Ndff (mg)} = \frac{39.4 \text{ x } 3.2}{100} = 1.3$$

$$\text{NUE (\%)} = \frac{\text{Ndff (mg) x 100}}{\text{Nt in administered labelled food}}$$

$$\text{NUE (\%)} = \frac{1.3 \text{ x } 100}{74.66} = 1.7$$

NUE in fish

Ndff (mg) in fish = Ndff Gills + Ndff fins + Ndff spleen + Ndff mouth + Ndff head + Ndff brain + Ndff pyloric caeca + Ndff esophagus content + Ndff stomach content + Ndff intestinal content + Ndff heart + Ndff esophagus + Ndff stomach + Ndff liver + Ndff intestine + anus + Ndff kidney + Ndff trunk

$$\text{Ndff (mg) in fish} = 67.11 \text{ mg}$$

$$\text{NUE (\%)} = \frac{\text{Ndff (mg)in fish x 100}}{\text{Nt in administered labelled food}}$$

$$\text{NUE (\%)} = \frac{67.11 \text{ x } 100}{74.66} = 89.9\%$$
